# Exposure to Conspecific and Heterospecific Sex-Pheromones Modulates Gustatory Habituation in the Moth *Agrotis ipsilon*

**DOI:** 10.3389/fphys.2019.01518

**Published:** 2019-12-20

**Authors:** Camille Hostachy, Philippe Couzi, Guillaume Portemer, Melissa Hanafi-Portier, Meena Murmu, Nina Deisig, Matthieu Dacher

**Affiliations:** CNRS, INRA, IRD, Institute for Ecology and Environmental Sciences of Paris, Sorbonne Universite, Universite Paris Est Creteil, Paris, France

**Keywords:** insect, moth, gustatory perception, sugar responsiveness, non-associative learning, habituation, proboscis extension response, pheromone

## Abstract

In several insects, sex-pheromones are essential for reproduction and reproductive isolation. Pheromones generally elicit stereotyped behaviors. In moths, these are attraction to conspecific sex-pheromone sources and deterrence for heterospecific sex-pheromone. Contrasting with these innate behaviors, some results in social insects point toward effects of non-sex-pheromones on perception and learning. We report the effects of sex-pheromone pre-exposure on gustatory perception and habituation (a non-associative learning) in male *Agrotis ipsilon* moths, a non-social insect. We also studied the effect of Z5-decenyl acetate (Z5), a compound of the sex-pheromone of the related species *Agrotis segetum*. We hypothesized that conspecific sex-pheromone and Z5 would have opposite effects. Pre-exposure to either the conspecific sex-pheromone or Z5 lasted 15 min and was done either immediately or 24 h before the experiments, using their solvent alone (hexane) as control. In a sucrose responsiveness assay, pre-exposure to the conspecific sex-pheromone had no effect on the dose-response curve at either delays. By contrast, Z5 slightly improved sucrose responsiveness 15 min but not 24 h after pre-exposure. Interestingly, the conspecific sex-pheromone and Z5 had time-dependent effects on gustatory habituation: pre-exposing the moths with Z5 hindered learning after immediate but not 24-h pre-exposure, whereas pre-exposure to the conspecific sex-pheromone hindered learning at 24-h but not immediate pre-exposure. They did not have opposite effects. This is the first time a sex-pheromone is reported to affect learning in a non-social insect. The difference in modulation between conspecific sex-pheromone and Z5 suggests that con- and hetero-specific sex-pheromones act on plasticity through different cerebral pathways.

## Introduction

Males of many moths species display a stereotyped, very specific, and innate attraction response to the sex-pheromone released by conspecific females (Allison and Cardé, 2016a). They are also able to detect and avoid heterospecific sex-pheromones from related sympatric species, which favors reproductive isolation (Renou et al., 1996; Allison and Cardé, 2016b). These attraction/deterrence responses are crucial for moth reproduction. As these insects are exquisitely sensitive to their sex-pheromone, they have been studied a lot as models of odor specialist. Moreover, as many moth species are crop pests, behavioral manipulation by use of pheromones is an important tool for managing their populations (Cook et al., 2007; Witzgall et al., 2010; Cork, 2016; Evenden, 2016). Therefore, studying their responses to pheromones has also important applications. These features make moths important models to study how pheromone can trigger stereotyped behaviors.

While pheromones are classically described as elicitor of stereotyped behaviors, in bees and mammals non-sex-pheromones can modulate plasticity (Coureaud et al., 2006; Vergoz et al., 2007; Bredy and Barad, 2009; Jouhanneau et al., 2016). In particular, reports in bees point toward effects on gustatory learning and perception (Pankiw and Page, 2003; Urlacher et al., 2010; Baracchi et al., 2017). Many examples in moths show interactions between reproduction and gustatory perception and learning (Awmack and Leather, 2002; Geister et al., 2008; Shikano and Isman, 2009; Molleman, 2010; Minoli et al., 2012; Briscoe et al., 2013; Petit et al., 2015). Therefore, it is reasonable to hypothesize that sex-pheromones could also interact with the perception and learning of gustatory information in these insects.

Observing proboscis extension response (PER) is a convenient experimental procedure to investigate gustatory functions: when the insect antennae contact a sugar solution of sufficient concentration, a moth extends its proboscis (i.e., releases a PER). Thus, this reflex allows to assess sucrose-linked behaviors in restrained moths, which is relevant as sugars are their main food in nature (under the form of nectar). Using standardized dose-response curves, PER assays have been used to assess sucrose responsiveness in moths (Hostachy et al., 2019), as well as in bees (Scheiner et al., 2004a) and flies (Scheiner et al., 2004b). PER can also be used to train and study animals in a non-associative learning called gustatory habituation. In habituation, an animal decreases and stops its response to a stimulus, if this stimulus is ongoing or repeated and the animal does not undergo any consequence when stimulated; using a dishabituation test then confirms this response inhibition cannot be explained by fatigue or sensory adaptation (Rankin et al., 2009; Blumstein, 2016). Such dishabituation test consists in presenting of a more intense (or different) stimulus, and to observe whether the response to the original stimulus has been restored. It is easy to habituate the PER upon repeated presentations of a low-concentration sucrose solution on the antennae without feeding the animals: in that case, antennal stimulations cease eliciting the PER. Beyond moths, this protocol has been applied in bees and flies (Duerr and Quinn, 1982; Braun and Bicker, 1992; Dacher and Gauthier, 2008). Habituation can be modulated by non-sex-pheromones in bees (Baracchi et al., 2017). It is established that PER-based learning are relevant to natural conditions (Gerber et al., 1996; Sandoz et al., 2000; Chaffiol et al., 2005; Gil and De Marco, 2005; Riffell et al., 2013). Habituation leads to ignore irrelevant stimuli, which allows a reallocation of resources (Mennemeier et al., 1994; Dukas, 2002, 2004; Ramaswami, 2014; Turatto and Pascucci, 2016).

In this article, we took advantage of the PER to explore the links between reproduction and gustation in moths by assessing whether their sex-pheromone can modulate sucrose responsiveness and gustatory habituation. Male *Agrotis ipsilon* moths were thus pre-exposed to either their conspecific sex-pheromone or a heterospecific sex-pheromone compound of a related sympatric species (*Agrotis segetum*), which they can perceive and avoid (Renou et al., 1996). We then assessed whether this pre-exposure affects sucrose responsiveness or gustatory habituation either immediately or 24 h after exposition, as previous results suggest sex-pheromone elicits long-term effects (increase of the response to the sex-pheromone itself, Anderson et al., 2003, 2007). These pheromones were chosen with the idea that *A. ipsilon* sex-pheromone would be “positive” whereas *A. segetum* compound would be “negative” (as defined by Baracchi et al., 2017) as they are respectively attractive and deterrent for *A. ipsilon* males. Thus, we made the hypothesis that they would have opposite effects.

## Materials and Methods

### Animals

Male *Agrotis ipsilon* (Lepidoptera, Noctuidae) were reared in our breeding facilities in Versailles, France. Adults were kept in climatic chambers (22°C, 60–70% relative humidity) and under an inverted light-dark cycle (16 h of light, starting at 18 h) as they are nocturnal insects. They were used 5 days after emergence and were provided with water *ad libitum* instead of the sucrose solution normally offered as a food source. This 5-day starvation duration optimizes the responses to sucrose without making the animals weak (Hostachy et al., 2019). Moreover, at this age, they have reached the peak of their sensitivity to the sex-pheromone. Males and females were separated at the pupal stage, so that animals were naive for the sex-pheromone before pre-exposure.

Experiments were performed between 13 and 17 h (activity peak of the animals) under dim red light. Before 10 h, animals were restrained in small tubes (made with cut 1 ml pipette cones) and their position was secured with adhesive tape and a small ball of absorbing paper behind them, so that only their heads (including antennae and proboscis) protruded from the tube.

### Pheromone Pre-exposure

A behaviorally active blend consisting of Z7-dodecenyl-acetate, Z9-tetradecenyl-acetate, and Z11-hexadecenyl-acetate in a 4:1:4 ratio was used as conspecific sex-pheromone (Picimbon et al., 1997). The precise ratio of each blend was checked by gas chromatography. We also used Z5-decenyl acetate (Z5), one of the main compounds of the sex-pheromone of *Agrotis segetum*. Both the conspecific sex-pheromone and heterospecific Z5 were diluted in hexane (10 ng/μl). Pheromonal compounds were purchased from Pherobank^1^ (Wijk bij Duurstede, Netherlands).

Pre-exposure was performed by positioning the moth during 15 min in a glass vial (2.5 cm diameter, 6 cm length) containing a small filter paper with 10 ng of conspecific sex-pheromone or Z5. This 15-min pre-exposure was performed either immediately or 24 h before performing sucrose responsiveness or gustatory habituation protocols. Moths were already restrained for the 15-min pre-exposure. The filter paper was prepared before by depositing 1 μl of conspecific sex-pheromone or Z5 in solution in hexane, waiting for hexane to vaporize, and then putting the paper into the vial. Control animals followed the same procedure except that the filter paper had 1 μl hexane without conspecific sex-pheromone or Z5.

### Sucrose Responsiveness

The standardized sucrose responsiveness assay was described for moths by Hostachy et al. (2019), adapting Scheiner’s protocol previously developed for bees and flies (Scheiner et al., 2004a,b). This assay consisted in presenting to each moth a succession of sucrose solutions of logarithmically increasing concentrations (0, 0.1, 0.3, 1, 3, 10, 30% and again 0%, weight/weight). Each presentation (every 10 min) consisted in touching both antennae during 1–4 s with a toothpick imbibed with one of the sucrose solution, and to record whether a PER was elicited; animals were not fed. A dose-response curve was then obtained, displaying for each sucrose concentration the PER rate (i.e., the percentage of moth exhibiting a PER in response to the antennal stimulation with the sucrose solution). Animals spontaneously responding to the initial water presentation were not kept in the analysis (although keeping them would not change the conclusions, data not shown). In these experimental conditions, the sugar presentations can be considered as independent of each other. Indeed, it was previously observed that presenting the solutions in a random order did not change the PER rates (Hostachy et al., 2019).

### Gustatory Habituation

The habituation protocol consisted in presenting a 3% (weight/weight) sucrose solution for 1–4 s every 10 s on both antennae without feeding the animal. The habituation criterion was defined as failing to release a PER to four consecutive presentations. Moths reaching this criterion were then submitted to a dishabituation test, consisting in presenting a 66% (weight/weight) sucrose solution and then the initial 3% solution. This restored the PER in most of these animals, which were then considered habituated. Resuming the response indicates fatigue or sensory adaptation cannot explain their reaching the habituation criterion (Rankin et al., 2009). Animals not responding to the first 3% presentation or not dishabituating were removed from the analysis. However, the proportions of such animals were compared across the treatments. Otherwise, animals reaching 30 sucrose presentations without habituating were considered as non-habituated. Sucrose concentrations and inter-trial interval were established after preliminary experiments and allows to clearly and reliably notice the occurrence of habituation while keeping the protocol practicable. While more moths can be habituated by going beyond 30 trials (i.e., 5 min per animal), this also increases the duration of the experiment without making the analysis more sensitive.

### Data Analysis

Statistics were performed with R 3.6, using an *α* risk of 5%. In sucrose responsiveness experiments, *χ*^2^ was used to compare PER rates of different groups for each sugar concentration. Fisher’s exact test was used when *χ*^2^’s assumption were not respected (i.e., when Cochran’s criterion was not met). Subsequent pairwise comparisons were made when the global test was significant using the same test (*χ*^2^ or Fisher’s exact test) and adjusting *p* for multiple comparisons with Holm’s method.

For habituation experiments, survival analyses were made using Cox regression (a survival analysis) to compare the probability of habituating between Z5- or conspecific sex-pheromone-treated animals and the control group (hexane-treated animals). Validity of the proportional hazard hypothesis in the Cox regression was confirmed by the Schoenfeld test (data not shown). Cox regression is particularly appropriate to analyze such data, as it can take into account the fact not all animals reach the habituation criterion. To compare the proportions of animals not responding to the first presentation of the 3% sucrose solution among all the animals initially tested, we used *χ*^2^ or Fisher’s exact test. Similarly, these tests were used to compare across the treatments the proportion of animals failing to dishabituate among those reaching the habituation criterion.

## Results

### Sucrose Responsiveness After Sex Pheromone Pre-exposure

**Figure 1:**
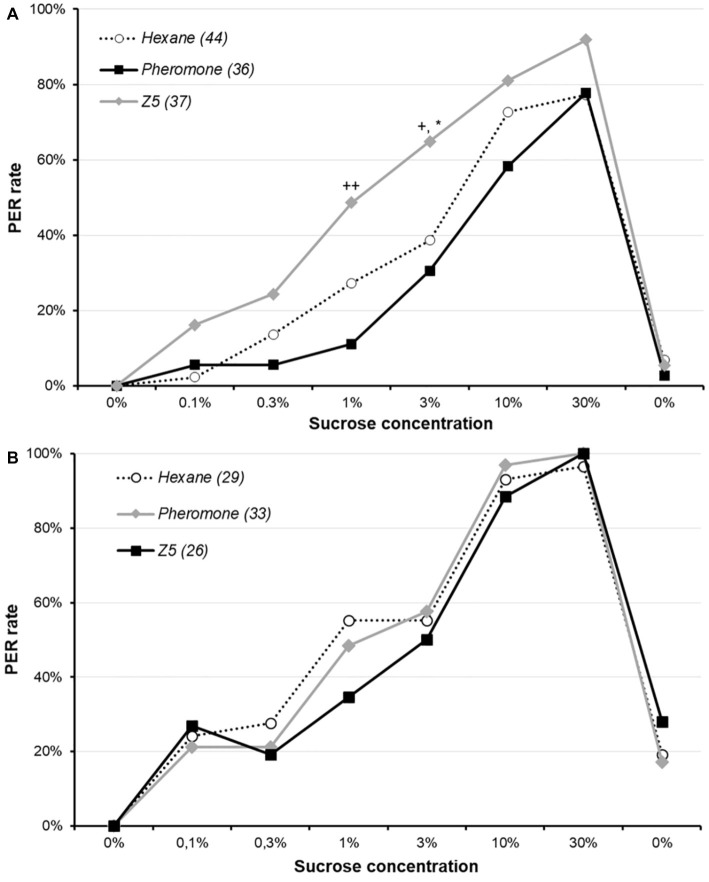
Effect of sex-pheromone on sucrose responsiveness in male *Agrotis ipsilon*. The x-axis reports the successive sucrose solutions, and the y-axis the PER rate (i.e., percentage of animals responding by a PER). Each curve corresponds to a treatment (i.e., pre-exposure to hexane, pheromone or Z5), and values in parenthesis are the sample sizes. In part **A**, animals were exposed to either hexane, conspecific sex-pheromone or Z5 during 15 min before undertaking the sucrose responsiveness assay. In part **B**, the pre-exposure was done for 15 min 24 h before the sucrose responsiveness assay. Stars denote a significant difference between Z5 and hexane (*χ^2^, adjusted *p* < 0.050), and crosses between Z5 and conspecific sex-pheromone (*χ*^2^; +: adjusted *p* < 0.050; ++: adjusted *p* < 0.050).

Moths were pre-exposed for 15 min to either conspecific sex-pheromone, Z5 or hexane immediately before having their sucrose responsiveness assessed (Figure 1A). Animals exposed to Z5 had significantly higher response rates than those exposed to sex-pheromone or hexane for 3% sucrose concentrations (*χ*^2^, adjusted *p* ≤ 0.037), whereas these two groups did not differ (*χ*^2^, adjusted *p* = 0.451). Similarly, Z5- and pheromone-exposed animals differed for 1% sucrose concentrations (*χ*^2^, adjusted *p* = 0.001), although neither of them differed from hexane-exposed animals (*χ*^2^, adjusted *p* = 0.094 for both comparisons). This suggest a short-term Z5 pre-exposure somewhat increases sucrose responsiveness.

The same experiment was performed with a 24 h delay between pre-exposure and the assay (Figure 1B). Response rates of three treatments did not significantly differ for any sucrose concentration (*χ*^2^ or Fisher’s exact test: *p* ≥ 0.301). This indicates a long-term exposure to Z5 or conspecific sex-pheromone does not affect sucrose responsiveness.

### Gustatory Habituation After Sex-Pheromone Pre-exposure

**Figure 2:**
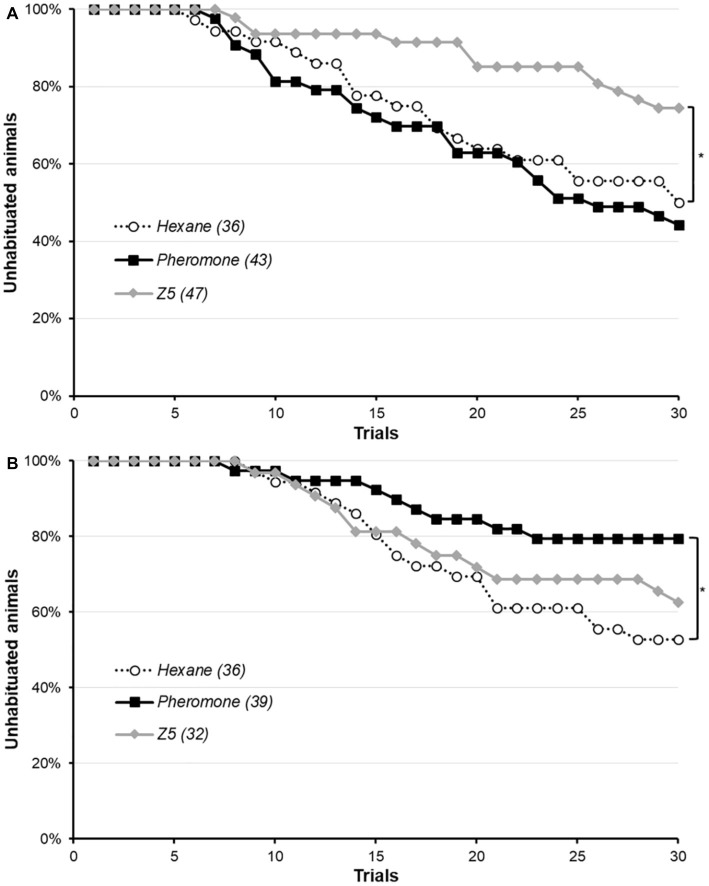
Effect of conspecific sex-pheromone and Z5 on gustatory habituation in male *Agrotis ipsilon*. The x-axis shows the trials in the habituation protocol, and the y-axis the proportion of unhabituated moths, which starts at 100% and then decrease as more and more moths reach the habituation criterion. Each curve corresponds to a treatment (i.e., pre-exposure to hexane, conspecific sex-pheromone or Z5), and values in parenthesis are the sample sizes. In part **A**, the pre-exposure was done immediately before the habituation protocol and in part **B**, it was done 24 h before the habituation protocol. Significant difference with the control hexane group are denoted by a star (^*^: Cox regression, *p* < 0.050).

The gustatory habituation protocol was performed immediately after a 15 min pre-exposure of conspecific sex-pheromone, Z5 or hexane performed as previously (Figure 2A). There was no difference between the three groups for the initial PER rate to the 3% sucrose solution used in the protocol (*χ*^2^, *p* = 0.165). This was unexpected, as we previously observed Z5 enhance the PER rate for this concentration. Animals not responding to this initial stimulation were discarded. Among animals reaching the habituation criterion at the end of the protocol, some were not able to dishabituate upon presentation of a 66% sucrose solution followed by a 3% sucrose solution, and were not used in the analysis. The proportion was the same across the three groups (*χ*^2^, *p* = 0.060). Moths pre-exposed to their sex-pheromone did not differ from the control (Cox regression, *p* = 0.578), whereas pre-exposure to Z5 significantly hindered habituation (Cox regression, *p* = 0.018) as less animals had reached the habituation criterion at the end of the protocol. Interestingly, the opposite effect was observed for a pre-exposure 24 h before the protocol (Figure 2B): conspecific sex-pheromone significantly reduced habituation, whereas Z5 was not significantly different from the control (Cox regression: sex-pheromone, *p* = 0.021; Z5, *p* = 0.460). In this experiment, as expected the PER rate was the same in the three groups for the initial 3% sucrose solution (*χ*^2^, *p* = 0.152), but the dishabituation rate was not the same (Fisher’s exact test, *p* = 0.037): indeed, hexane- and sex-pheromone exposed animals always dishabituated, but not all Z5-exposed. Animals failing to dishabituate were not included in Figure 2B, nor in the analysis, and were a minority (for hexane, 17 were habituated and 19 not habituated, for a total of 36 moths and none failed to dishabituate; for conspecific sex-pheromone, 8 were habituated 31 not habituated, for a total of 39 moths and none failed to dishabituate; for Z5, 16 moths reached the habituation criterion but 4 of them did not dishabituate and were excluded, leaving 12 habituated moths and 20 not habituated for a total of 32 moths).

## Discussion

Responsiveness to sucrose was not modulated by a pre-exposition to conspecific sex-pheromone in male *A. ipsilon* moths. By contrast, the major pheromone component of a sympatric species (Z5) increased sucrose responsiveness but only at the short-term exposure. Furthermore, habituation, a form of non-associative learning was hindered by both pheromones, but at different delays: immediately but not 24 h after exposure for Z5, and 24 h but not immediately after exposure for the conspecific sex-pheromone.

Experiments in bees and ants have shown that pheromone exposure modulates sucrose responsiveness and performance during learning, including gustatory habituation (Pankiw and Page, 2003; Vergoz et al., 2007; Urlacher et al., 2010; Minoli et al., 2012; Baracchi et al., 2017; Rossi et al., 2018). Overall, pre-exposure to pheromone sensitizes or desensitize insects (according to the “positive” or “negative” value of the pheromone, Baracchi et al., 2017), thereby affecting their sucrose responsiveness. In our experiments, exposition to the conspecific sex-pheromone did not affect sucrose responsiveness. Thus, an effect on habituation through a modulation of sucrose responsiveness is excluded. By contrast, the fact that Z5 increases sucrose responsiveness is consistent with its impairing habituation to sucrose (even though it did not initially decrease the PER rate in this experiment). A striking difference is the temporal difference of conspecific sex-pheromone and Z5: the later acts immediately but not after 1 day (except for a small effect on dishabituation rate), whereas sex-pheromone needs 1 day to have an effect and only affects habituation. This indicates that they act through different pathways.

The neurophysiological mechanisms underlying these effects of conspecific sex-pheromone and Z5 remain an open question. Such mechanisms could involve biogenic amines. Indeed, these neurotransmitters modulate responsiveness to sucrose and learning (Scheiner et al., 2006, 2014), and biogenic amines are involved in sex-pheromone’s actions (Duportets et al., 2010; Abrieux et al., 2014). A possible mechanism for pheromone effects would be that they could have a positive or negative valence for the animal: perceiving them as reward (conspecific sex-pheromone) or punishment (heterospecific sex-pheromone such as Z5) could explain their effect on learning (Coureaud et al., 2006; Vergoz et al., 2007; Jouhanneau et al., 2016; Baracchi et al., 2017). However, this is not what we observed; both conspecific sex-pheromone and Z5 had the same deterring effect on habituation rather than opposite ones.

Pheromonal modulation of gustatory habituation is an interaction between reproductive and feeding functions. Impairing gustatory habituation could mean moths are less prone to ignore food. In that frame, Z5 would do more than just prevent mating with incompatible females and would inform on the presence of competitors for food. In turn, habituation would be hindered (and sucrose sensitivity improved) upon detecting them, in order to promote eating in the context of competition for food. By contrast, this would not make sense anymore 24 h after. Similarly, conspecific sex-pheromone would indicate the presence of mates in the environment. While habituation should be maintained immediately after perceiving the pheromone, in the absence of mating 24 h after exposure food should not be ignored so as to gather resources to continue mate-searching. This is consistent with the observation that sucrose can sensitize the response to sex-pheromone (Minoli et al., 2012).

These hypotheses are arguably bold, but are testable as they make specific and strong predictions: presenting conspecific sex-pheromone both 24 h and immediately before mating would improve habituation, as the male would focus on mating rather than feeding.

To further investigate the functional relationships between feeding and reproduction, it would be interesting to assess the amount of sucrose consumed by *A. ipsilon* males having differing experiences with conspecific females (e.g., exposure, mating) and/or sex-pheromones (Sokolowski and Abramson, 2010). The same experiments could also be done with exposure to females of *A. segetum* and Z5. An interesting feature of males in some Lepidoptera species is puddling, i.e., drinking brackish water to get sodium needed for gamete formation (Smedley and Eisner, 1996; Boggs and Dau, 2004; Watanabe and Kamikubo, 2005; Molleman, 2010). Owing to our observations, it would also be relevant to assess whether puddling is modulated by exposure to sex-pheromones.

## Data Availability Statement

All datasets generated for this study are included in the article.

## Author Contributions

CH, ND, and MD designed the experiments. CH, PC, GP, MH-P, and MM performed the experiments. CH and MD prepared the figures and analyzed the data. MD wrote the first draft of the manuscript, and all authors contributed to manuscript revision, read and approved the submitted version.

### Conflict of Interest

The authors declare that the research was conducted in the absence of any commercial or financial relationships that could be construed as a potential conflict of interest.

## Footnotes

^1^http://www.pherobank.com

## Abbreviations

PER: Proboscis extension response
Z5: Z5-decenyl acetate.
